# Dysphagia presentation and management following COVID-19: an acute care tertiary centre experience

**DOI:** 10.1017/S0022215120002443

**Published:** 2020-11-10

**Authors:** C Dawson, R Capewell, S Ellis, S Matthews, S Adamson, M Wood, L Fitch, K Reid, M Shaw, J Wheeler, P Pracy, P Nankivell, N Sharma

**Affiliations:** 1 Department of Speech and Language Therapy, Queen Elizabeth Hospital, Birmingham, UK; 2 Department of Otolaryngology, Queen Elizabeth Hospital, Birmingham, UK; 3 Institute of Cancer and Genomic Sciences, University of Birmingham, UK

**Keywords:** Dysphagia, COVID-19, Coronavirus, Swallowing, Rehabilitation

## Abstract

**Objectives:**

As the pathophysiology of COVID-19 emerges, this paper describes dysphagia as a sequela of the disease, including its diagnosis and management, hypothesised causes, symptomatology in relation to viral progression, and concurrent variables such as intubation, tracheostomy and delirium, at a tertiary UK hospital.

**Results:**

During the first wave of the COVID-19 pandemic, 208 out of 736 patients (28.9 per cent) admitted to our institution with SARS-CoV-2 were referred for swallow assessment. Of the 208 patients, 102 were admitted to the intensive treatment unit for mechanical ventilation support, of which 82 were tracheostomised. The majority of patients regained near normal swallow function prior to discharge, regardless of intubation duration or tracheostomy status.

**Conclusion:**

Dysphagia is prevalent in patients admitted either to the intensive treatment unit or the ward with COVID-19 related respiratory issues. This paper describes the crucial role of intensive swallow rehabilitation to manage dysphagia associated with this disease, including therapeutic respiratory weaning for those with a tracheostomy.

## Introduction

Severe acute respiratory syndrome coronavirus 2 (SARS-CoV-2) has affected over 24 million people globally, with over 327 643 cases reported in the UK.^1^ Patients with COVID-19 may have multi-organ system pathology, and as a result often require prolonged periods of rehabilitation. The role of swallow rehabilitation in patients with COVID-19 has not been reported, despite many of these patients presenting with dysphagia in acute care hospitals.

Dysphagia is associated with compromised patient outcomes, including aspiration pneumonia, malnutrition, increased length of admission and higher mortality.^2–5^ Dysphagia, its management and the hypothesised causes as a result of COVID-19 are yet to be understood. No prospective studies have explored this specific sequela. In addition, at the time of writing, practice guidelines are limited to expert consensus publications, without patient level data or outcomes. As patients are optimised on the Intensive Treatment Unit (ITU)/ward setting and survive COVID-19, the impact of intubation and extubation, proning, tracheostomy, critical illness and delirium become apparent. These patients have a significant burden of dysphagia requiring substantial intervention, in a setting made more complex by the novel symptomatology of an emerging disease, and the requirement to deliver this care in personal protective equipment (PPE).

We collected and analysed our single centre outcomes to define the pattern of recovery in our centre, to build the knowledge base to better understand the dysphagia presentation with COVID-19, and to support other clinicians in the field to optimise dysphagia outcomes for patients following COVID-19.

### Aims

This paper describes the dysphagia and functional swallow outcomes during recovery from COVID-19 in a large cohort of patients. Our primary outcomes included: prevalence of dysphagia associated with COVID-19; time taken to commence oral diet and or fluids; and the relationship between tracheostomy and intubation on dysphagia and recovery. Secondary study objectives included reflecting on key issues associated with managing this population, providing a narrative review of our experience.

## Materials and methods

The Standards for Quality Improvement Reporting Excellence (‘SQUIRE’ 2.0) reporting structure^6^ was followed for this evaluation.

### Ethical approval

This study was a prospective service evaluation using anonymised routinely collected data. As per the UK Research and Innovation and Health Research Authority decision tool,^7^ ethical approval was not required. Data are available only on request because of privacy and ethical restrictions.

### Setting and patient selection

All patients admitted to the Queen Elizabeth Hospital Birmingham with SARS-CoV-2 referred to the Speech and Language Therapy (SLT) team between 21/3/2020 until 21/5/2020 as a result of clinical signs of dysphagia identified by staff members were included. These included, but were not limited to, coughing during or after swallow, pain or difficulty swallowing, reduced oral intake, or presumed aspiration events following chest x-ray. All data were recorded contemporaneously on to the hospital electronic systems and then retrospectively analysed. SARS-CoV-2 positivity was confirmed by real-time reverse transcription (rtPCR) nasopharyngeal swab assay or non-directed bronchial lavage/aspirate.

### Swallow assessment and therapy protocol

During the COVID-19 pandemic, national and international institutions advised against the use of nasendoscopy given its potential stimulus as an aerosol-generating procedure (AGP).^8^ As such, only specific patients with complex presentations including pervasive voice change and dysphagia that showed no sign of improvement with compensatory techniques underwent fibre optic endoscopic evaluation of swallow (FEES). In place of nasendoscopy, an institution-specific standardised protocol of clinical bedside and perceptual skills was used to assess swallow, and the findings were documented by the speech and language therapy team. Full PPE (including gown, visor, gloves and filtering facepiece code 3 (FFP3) mask) was worn during dysphagia assessments. In order to reduce the risk of secondary bacterial chest infections due to aspiration post-COVID-19, intensive rehabilitation strategies were utilised to avoid further chest compromise.

### Assessment

Face-to-face swallow assessment included oro-motor examination to explore motor function (strength, speed and range of movement) of intra-oral musculature, including cranial nerve examination. Voice quality was also assessed but the data are not reported here. The International Dysphagia Diet Standardisation Initiative^9^ was used to describe the level of food or fluid used during the assessment of swallow (Table 1). Observation of the patient swallowing, perceptual analysis of any overt clinical signs of dysphagia and use of laryngeal palpation were optional for therapists involved in care.

**Table 1.** International Dysphagia Diet Standardisation Initiative levels of food and fluid intake for patients with dysphagia

Level of fluid	Description	Level of diet	Description
NBM		NBM	
Level 0	Thin fluid		
Level 1	Slightly thick		
Level 2	Mildly thick		
Level 3	Moderately thick	Level 3	Liquidised
Level 4	Extremely thick	Level 4	Pureed
		Level 5	Minced & moist
		Level 6	Soft & bite-sized
		Level 7	Regular or easy to chew

NBM = nil by mouth

### Therapy intervention

For patients presenting with signs of distress or difficulty managing increasing textures of diet complexity, therapy and compensation strategies included: exercise prescription, postural adaptations, practice swallows, augmentation of texture or complexity of diet and or fluid if required, portion volume control, adaptations to environmental factors (such as feeding support for patients with upper limb weakness), and reducing distractions. This was undertaken in a multidisciplinary context, with dietetic, physiotherapy and occupational therapy teams working collaboratively to titrate nasogastric tube feeding and oral intake, optimise respiratory status, and manage positioning for assessment and meal times.

### Outcomes

Principle outcome measures were: length of stay on the ITU, timing of speech and language therapy assessment, timing of oral intake commencement, and swallow competence at various timepoints.

### Statistics

Data were analysed using IBM SPSS Statistics® software (version 26). The distribution of continuous variables was tested using the one-sample Kolmogorov–Smirnov test. If normal distribution was shown, variables were presented as mean (standard deviation (SD)). Means of two continuous, normally distributed variables were compared using the independent samples student's *t*-test. Frequencies of categorical variables were compared using the chi-square test. A *p*-value of less than 0.05 was considered significant. Pearson's correlation was used to assess significance between paired, continuous variables.

## Results

During the first surge of COVID-19 (March to May 2020), approximately 1700 patients were admitted to the Queen Elizabeth Hospital, Birmingham, UK, following a positive test, of which 720 were admitted for over 3 days. Within this group, 204 out of 720 (28.3%) were admitted to the ITU for ventilatory support. In total, 208 out of 720 (28.9%) patients were referred to the speech and language therapy team, which included 102 of the 204 (50%) of the ITU cohort. Tracheostomy was performed in 82 (80%) of the ITU group referred to the speech and language therapy team, with the rest being primarily extubated. Mean patient age was 67.6 (SD = 17.6) years.

### Referral and initial assessment

All patients were assessed by the speech and language therapy team within 24 hours of a referral as part of our 7-day service. Mean (SD) time to referral was 7 (11.4) days after admission for patients managed solely on the ward. Within the ITU endotracheal tube cohort, mean (SD) time to referral to the speech and language therapy team was: 3 (1.6) days following extubation, 11 (5.3) days from date of tracheostomy for patients with a tracheostomy and 10 (5.2) days from the date of sedation being discontinued.

The main presenting features were: delirium – hyperactive or hypoactive presentation; laryngeal compromise – vocal cord not fold palsy and or laryngeal oedema; respiratory swallow co-ordination challenge; burden of secretions and constant expectoration; and fatigue.

Of the patients who were initially assessed on the ITU, 33% (19 out of 58) started on an augmented diet or fluid, and 67% (39 out of 58) were recommended to be nil by mouth. Only one patient was able to manage a level 7 (regular or easy to chew) diet at initial assessment on the ITU. For ward patients, 22% (23 out of 106) were recommended to be nil by mouth at initial assessment; however, 29% (31 out of 106) were able to be immediately placed on a level 7 diet. Of the patients surviving to begin oral intake (*n* = 193), 20% and 76% began with altered fluids and diet, respectively. ITU patients with a tracheostomy were more likely to begin an altered diet (Table 2) than those without (87% *vs* 59%, *p* = 0.003).

**Table 2.** Patients recommended for altered diet and fluids on commencement of oral intake

Patient group	Patients commencing altered fluids (% (*n*))	Patients commencing altered diet (% (*n*))
All patients	21 (39/193)	76 (145/193)
Ward cohort	36 (33/93)	70 (65/92)
ITU cohort	7 (7/100)	81 (81/100)
ETT alone	18 (4/22)	59 (13/22)
Tracheostomy	4 (3/78)	87 (68/78)

ITU = intensive treatment unit; ETT = endotracheal tube

For the majority of patients, dysphagia was complicated by: delirium, the use of sedation, frequent expectoration of high-volume secretions and significant fatigue. Holistic symptom burden influenced dysphagia clinical decision-making and oral intake guidance as much as overt symptoms of dysphagia did, specifically regarding recommendations to remain nil by mouth, which was frequently reviewed in the early phase assessment. A subset of five patients presented with vocal cord not fold palsies, confirmed on nasendoscopy.

### Oral intake commencement

Mean (SD) time to starting oral intake from extubation for the endotracheal tube only group was 5.3 (2.3) days. For patients with a tracheostomy, mean (SD) time to starting oral intake from the date of tracheostomy insertion was 14.8 (6.6) days, and from the cessation of sedation it was 13.0 (6.0) days. Mean (SD) time from intubation to commencing oral intake was longer for the tracheostomy group than the endotracheal tube group (28.0 (8.5) days *vs* 15.8 (6.2) days, respectively; *p* < 0.001).

**Fig. 1.**
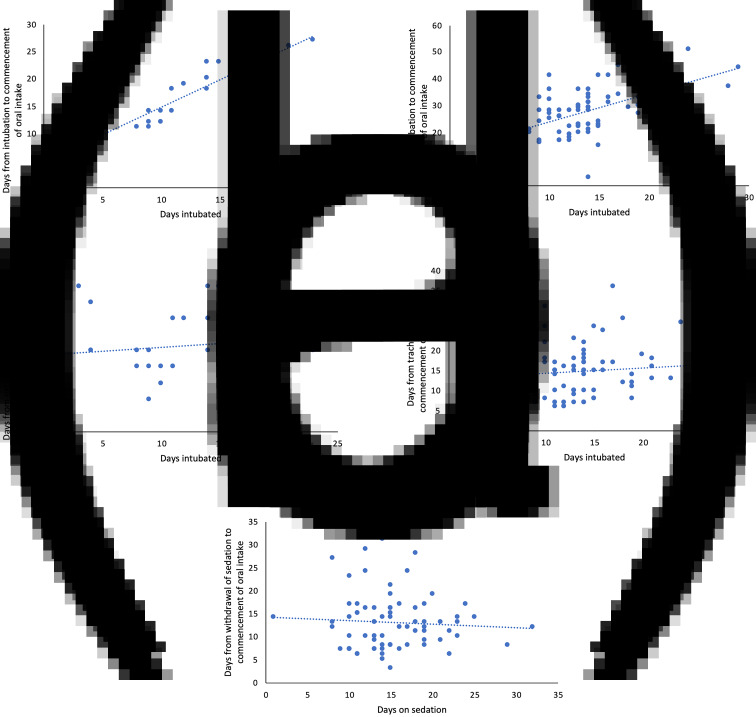
There was a positive correlation between the number of days a patient was intubated and the number of days from intubation to commencing oral intake for both (a) the endotracheal tube group (R^2^ = 0.84, *p* < 0.01) and (b) the tracheostomy group (R^2^ = 0.31, *p* < 0.01). The mean (standard deviation) time from extubation to oral intake (5.2 (2.3) days) or tracheostomy insertion to oral intake (14.7 (6.5) days) was not associated with duration of intubation for either (c) the endotracheal tube group (R^2^ = 0.01, *p* = 0.63) or (d) the tracheostomy group (R^2^ = 0.01, *p* = 0.44). (e) For the tracheostomy group, there was no correlation between the number of days on sedation and the period of time from stopping sedation to starting oral intake (R^2^ = 0.00, *p* = 0.58).

There was a significant positive correlation between the number of days a patient was intubated and the number of days from intubation to commencing oral intake for both the endotracheal tube group (R^2^ = 0.84, *p* < 0.01; Figure 1a) and the tracheostomy group (R^2^ = 0.31, *p* < 0.01; Figure 1b). The mean time from extubation to oral intake (5.2 (2.3) days) or tracheostomy insertion to oral intake (14.7 (6.5) days) was not associated with intubation duration for either the endotracheal tube patients (R^2^ = 0.01, *p* = 0.63; Figure 1c) or the tracheostomy patients (R^2^ = 0.01, *p* = 0.44; Figure 1d). For patients who underwent tracheostomy, mean time to starting oral intake after stopping sedation was 13.0 (6.0) days; there was no correlation between the number of days on sedation (15.2 (5.2) days) and the period of time from stopping sedation to starting oral intake (R^2^ = 0.00, *p* = 0.58) (Figure 1e). During this period, patients received swallow therapy and targeted rehabilitation to improve swallow competence.

### Progression through therapy

**Fig. 2.**
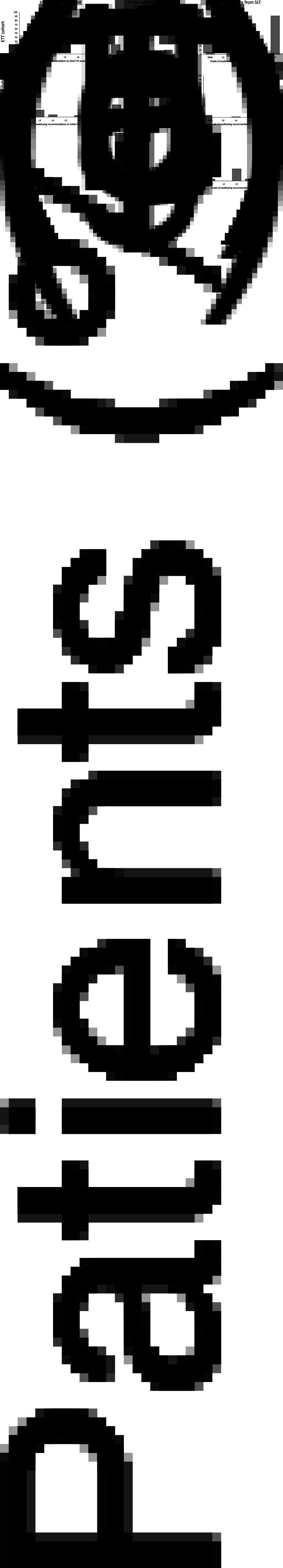
The degree of altered diet recommendations for patients at each stage – based on initial assessments on the initial intensive treatment unit (ITU) and on the ward, and assessment at discharge from speech and language therapy (SLT) – for the endotracheal tube (ETT) cohort (a–c respectively), the tracheostomy cohort (d–f respectively) and the ward cohort (g & h respectively). NBM = nil by mouth; L = level of diet (see Table 1)

The degree of altered diet recommendations for patients at each stage is shown in Figure 2. This demonstrates a progressive and linear improvement for the cohort from the ITU to discharge from the speech and language therapy team.

One patient remained nil by mouth because of neurological compromise, another two because of pervasive laryngeal issues; the remaining patients were still receiving therapy. For surviving patients managed only at ward level, 47% (21 out of 45) and 63% (28 out of 45) tolerated level 7 (regular or easy to chew) and level 6/7 (soft and bite-sized to regular) diets respectively at discharge from our acute care speech and language therapy team; many were transferred to community rehabilitation beds before they had finished speech and language therapy swallow rehabilitation. No patients were readmitted or presented with new aspiration pneumonia following discharge.

On average, the speech and language therapy team provided clinical interventions to patients for 8.6 days. This increased to 11.3 days for those who underwent endotracheal tube intubation alone, and was highest for those who had a tracheostomy (12.9 days, *p* = 0.002; average 5.3 sessions, *p* = 0.02); ITU patients as a whole required more input than ward patients (*p* < 0.001) (Table 3). These data do not include multidisciplinary team (MDT) discussion or bedside consultations without direct therapy, which was a continuous ongoing process for all patients.

**Table 3.** Degree of SLT input

Patient group	Total SLT duration (mean (SD); days)	SLT sessions per patient (mean (SD); *n*)
All patients	8.6 (9.4)	4 (2.8)
Ward cohort	5.8 (7.7)	3.2 (2.6)
ITU cohort	11.3 (10.1)	4.9 (2.8)
ETT alone	5.7 (5.8)	3.7 (2.3)
Tracheostomy	12.9 (10.5)	5.3 (2.9)

SLT = speech and language therapy; SD = standard deviation; ITU = intensive treatment unit; ETT = endotracheal tube

## Discussion

This paper describes the swallow outcomes for the largest published cohort of patients diagnosed with COVID-19. These patients had a high prevalence of dysphagia, of almost 30%, requiring significant therapeutic intervention. In our experience, with targeted therapy most patients will return to their baseline swallow function before discharge from hospital.

Patients presented with multi-factorial dysphagia, and whilst similar patterns of clinical presentation such as oral phase dysphagia were identified, each individual had a different hierarchy of clinical issues. These features individually or concurrently impacted on swallow competence, and required targeted rehabilitation from the speech and language therapy team. Most patients required modified diets to manage oral phase dysphagia, with some requiring a short period of thickened fluids to support oral control due to fatigue, delirium and poor lip closure. Thickener is not frequently used in our institution, with postural advice and exercise therapy being preferred; however, fluid thickened to a level 1 (slightly thick) consistency was effective for a small group of patients for a limited time as oral intake was reinitiated.

Patients with a tracheostomy required a greater total number of therapy sessions with the speech and language therapy team than patients who were admitted to the ward without an ITU stay. There was no correlation between duration of intubation and the length of time needed to commence oral intake after extubation or decannulation. This highlights the importance of targeted and sustained swallow therapy for patients post-extubation, suggesting that, with optimal rehabilitation, most patients can regain near normal swallow function prior to discharge in this context. This requires investment in appropriately skilled and resourced speech and language therapy teams.

Patients presenting with dysphagia post-tracheostomy required intensive therapy to support weaning to decannulation. This took place in a designated COVID-19 AGP ITU, ward, bay or side room, and staff wore appropriate PPE. Where clinically indicated, a number of measures were employed (cuff deflation, use of fenestrated inner tubes, downsizing of tracheostomy tubes, and use of speaking valves, to support laryngeal function or sensation, and to enhance expectoration), which some guidelines have advised avoiding to reduce infection potential.^10,11^ Mucolytics and secretion-drying agents were not used; patients were prescribed saline nebulisers, administered four times a day via the tracheostomy mask, to ensure the secretions remained hydrated. All but one patient who underwent tracheostomy were decannulated at the time of publication. One-third of patients required therapeutic interventions such as tracheostomy tube downsizing with fenestration, to improve laryngeal function.^12^

The psychosocial impact of COVID-19 was significant for patients. Unable to see family members, being treated by teams in full PPE and, for many, experiencing delirium ITU was traumatic.

People of Black, Asian and minority ethnic groups have a higher mortality rate from COVID-19 than white patients, with greater severity of disease, as reflected by a higher proportion of such patients with confirmed COVID-19 requiring invasive ventilation and other organ support than white patients.^13,14^ Our tertiary centre is located in Birmingham in the UK, a culturally and ethnically diverse area, with a high rate of admissions to hospital.^15^ For some, English was their second or third language, making communication with clinical teams difficult, with no easy access to translating services within infectious areas. We used novel approaches such as smart phones and multilingual staff members to support communication. The first wave of COVID-19 happened during the Islamic holy month of Ramadan, when many patients would have ordinarily been observing prayer and reflection whilst fasting during daylight hours; this made oral diet re-initiation and therapy particularly challenging, highlighting a departure from normal life, family, and their religious and cultural beliefs. Whilst time was pressured, personalised therapeutic space from the MDT was fundamental to holistic recovery.

### Recommendations

The consensus guidelines that have emerged provide easy access overviews for clinical teams; however, we suggest they are implemented with well-established clinical protocols and experienced clinicians in order to manage dysphagia in this context. We provided intensive therapy for the COVID-19 cohort with high level acuity and clinical needs, presentations of which we had not encountered before in such high numbers. We hypothesise that our positive clinical outcomes were influenced by the intensive swallow rehabilitation and tracheostomy care provided to this complex cohort of patients. We therefore recommend a whole systems approach to managing dysphagia associated with COVID-19, including medical, surgical, nursing, therapy and education teams.

### Limitations

Because of restrictions around AGPs, we could not differentially diagnose dysphagia and aspiration with instrumentation, nor could we examine the upper airway in detail to understand the mechanism of injury. This was a context rather than design limitation. Despite the caution that must be recognised in drawing conclusions from a non-randomised service evaluation, we present our experience in this large cohort and our narrative as a contribution to the literature following the first wave of the COVID-19 pandemic. We hope that this will support other acute care teams in the event of future surges to manage these complex sequelae.

• The prevalence of dysphagia is high in patients admitted with COVID-19, which is an important facet of rehabilitation needs
• Dysphagia was multi-factorial, and co-existed with delirium, fatigue and difficulty achieving effective breathe/swallow co-ordination
• Patients with a tracheostomy following COVID-19 required greater total therapy sessions from the Speech and Language Therapist (SLT)
• Acute trusts require robust, well established and appropriately funded SLT teams to provide timely, effective rehabilitation for patients with complex upper airway compromise following COVID-19 and acute admission
• A whole systems approach from an integrated multi-disciplinary team is required to manage concurrent and complex aetiology associated with COVID-19 and an acute admission
• With intensive therapy, most patients can regain normal swallow function following COVID-19

## Conclusion

This paper describes the swallow outcomes of the largest consecutive cohort of patients diagnosed with COVID-19; it suggests that by using intensive and targeted therapy, many patients can regain normal or near normal swallow function following intubation and tracheostomy, and liberation from tracheostomy. We hope that these data will provide guidance to other units to facilitate efficient flow through intensive treatment units and hospitals in the event of subsequent surges of COVID-19.
